# Integrate QTL Mapping and Transcription Profiles Reveal Candidate Genes Regulating Flowering Time in *Brassica napus*

**DOI:** 10.3389/fpls.2022.904198

**Published:** 2022-06-28

**Authors:** Zigang Liu, Xiaoyun Dong, Guoqiang Zheng, Chunmei Xu, Jiaping Wei, Junmei Cui, Xiaodong Cao, Hui Li, Xinlin Fang, Ying Wang, Haiyan Tian

**Affiliations:** State Key Laboratory of Arid Land Crop Sciences, Gansu Agricultural University, Lanzhou, China

**Keywords:** flowering time, QTL mapping, RNA-seq, candidate genes, *Brassica napus*

## Abstract

Flowering at the proper time is an important part of acclimation to the ambient environment and season and maximizes the plant yield. To reveal the genetic architecture and molecular regulation of flowering time in oilseed rape (*Brassica napus*), we performed an RNA-seq analysis of the two parents after vernalization at low temperature and combined this with quantitative trait loci (QTL) mapping in an F_2_ population. A genetic linkage map that included 1,017 markers merged into 268 bins and covered 793.53 cM was constructed. Two QTLs associated with flowering time were detected in the F_2_ population. qFTA06 was the major QTL in the 7.06 Mb interval on chromosome A06 and accounted for 19.3% of the phenotypic variation. qFTC08 was located on chromosome C06 and accounted for 8.6% of the phenotypic variation. RNA-seq analysis revealed 4,626 differentially expressed genes (DEGs) between two parents during vernalization. Integration between QTL mapping and RNA-seq analysis revealed six candidate genes involved in the regulation of flowering time through the circadian clock/photoperiod, auxin and ABA hormone signal, and cold signal transduction and vernalization pathways. These results provide insights into the molecular genetic architecture of flowering time in *B. napus*.

## Highlights

- Two QTLs associated with flowering time were identified in *Brassica napus*.
- The integration of QTL mapping and RNA-seq data allowed for the detection of six candidate genes that play important roles in the regulation of flowering.

## Introduction

*Brassica napus* is a major oil crop and the largest source of high-quality vegetable oil worldwide. There are three types (spring, winter, and semi-winter), categorized by the amount of low-temperature exposure needed for the transition from vegetative growth to flowering. Semi-winter types require little exposure to the low temperatures and are planted primarily in the Yangtze River basin of southern China. Spring rapeseed is planted in the high latitude region of China, and winter rapeseed is planted in the low latitude region. Winter rapeseed has many advantages over spring rapeseed, including grain yield, seed quality, resistance to adversity, etc., however, winter rapeseed cannot survive winter in northern China due to extreme low temperatures (<−20°C). Several varieties (e.g., NTS57, Ganyou 4, Ganyou 221) were bred *via* distant cross to introduce the cold-tolerance of winter turnip rape (*B. rapa*) to winter oilseed rape, enabling these varieties to overwinter in northern China, which is important to alleviate the shortage of vegetable oil and prevent sand from blowing from the fallow fields. These cold-tolerant varieties require prolonged exposure to low temperatures to initiate flowering. Vernalization is critical for flowering, and therefore also to grain yield, adaptation, and survival of winter crops.

Insufficient exposure to low temperatures resulting in inadequate vernalization delays the flowering of winter-type crops. It is possible to evaluate the degree of vernalization and obtain phenotypic data that can then be used to detect QTLs related to the vernalization of winter-type crops. A recombinant inbred line (RIL) wheat population was used to identify 15 QTLs linked to vernalization on nine chromosomes, and these QTLs accounted for 17 to 46% of phenotypic variation (Li et al., 2020). In total, five QTLs in four linkage groups accounted for 5.4 to 28.0% of the phenotypic variation in vernalization in an F_2_ population derived from two varieties of perennial ryegrass (Jensen et al., 2005). In the rapeseed, flowering time was used to evaluate vernalization in 127 RIL population lines and identify QTLs linked to vernalization. In total, two QTLs (dtf7.1 and dtf1.1) in two linkage groups accounted for 53.1 and 8.3% of the phenotypic variation, respectively (Lee et al., 2021). One double haploid (DH) population was used to identify five consensus QTLs in the three environments, including two major QTLs located on chromosomes A03 and C08, respectively (Lee et al., 2021). Raman et al. (2012) used the flowering time to detect > 20 QTLs, including seven related to vernalization that accounted for 59.4% of the phenotypic variation. Four indexes of phenotype in the DH population in six environments were used to identify QTLs, and two vernalization QTLs in chromosomes A02 and A07 were identified (Shen et al., 2018). F_2_ progeny and an F_2:3_ population were used to identify 10 QTLs related to flowering time in four environments, one of which may be a novel QTL on chromosome A09 (Xu et al., 2020).

Several cold-tolerant *B. napus* varieties that can survive below −25.6°C were bred in our laboratory. These strong winter types require more exposure to low temperatures for vernalization than do semi-winter and winter types, and this suggests that novel QTLs are responsible for regulating vernalization. In addition, if flowering time can be efficiently regulated using artificial measures, heterosis between two ecotypes (spring and strong winter types) can be exploited, and this is crucial for yield breeding of *B. napus*. Therefore, F_2_ mapping of a spring-ecotype population derived from NTS57 and CY12 was used to detect QTLs related to the flowering time and vernalization for efficient breeding applications.

## Materials and Methods

An F_2_ population was generated from a cross between two *B. napus* varieties, NTS57 (late-flowering, strong winter-type) and CY12 (early-flowering, spring-type), consisting of 174 individuals for QTL mapping. F_2_ seeds were first sowed into plugs with 45 holes filled with nutrient soil in a greenhouse at the Gansu Agriculture University in Lanzhou of China. One seed per hole was sowed to foster seedlings, and 4 weeks after sowing them, they were transplanted into 25 cm diameter pots. The temperature in the phytotron was maintained in the range from 18 to 22°C. About 9 weeks after sowing, seedlings were transplanted into a phytotron with a constant temperature of 4°C and an 8-h light period and placed for 40 days in the phytotron. After vernalization accomplishment, seedlings were returned to the greenhouse, and the growth situation of seedlings was observed every day to accurately record the date of the first flowering. Leaf of 9 weeks’ seedlings per F_2_ individual was harvested. Per sample was quick-frozen in liquid nitrogen, and stored at −80°C to extract DNA for genotype detection and construction of a genetic map.

Seeds of two parents, i.e., 17NTS57 and CY12, were sown into pots in the greenhouse with temperature ranging from 18 to 22°C. The 9 weeks seedlings of the two parents were harvested (T0), and after harvesting, seedlings were placed into the phytotron with a temperature as low as 4°C for vernalization. Leaves of two parents were harvested on 20 days (T1) and 40 days (T2), respectively, after initiating vernalization. Leaves harvested were quick-frozen and stored at −80°C to extract RNA for transcriptome and qRT-PCR analysis.

### Calculation of the Number of Days of First Flowering

Flowering time (FT), i.e., the number of days from sowing to the first flowering is a phenotypic date using QTL analysis, which was calculated using the following formula: FT = the date of first flowering from the date of sowing.

### DNA Extraction and Targeted Sequencing

Following the protocols of Doyle and Doyle (1990), genomic DNA from the young expanded leaves was extracted from each F_2_ plant. After quality assessment, each DNA sample was used to construct a library and perform targeted sequencing as described by GENOSEQ company (Wuhan, China). As sequence regions with known polymorphisms are attempted as targets for amplicon fragments, we utilized a total of 1,010 existing primer pairs from the previous SNP genotyping assays to target specific loci. These primers were mixed together in equal proportions. Mixed primers were diluted to ca. 54 nM aliquots and were used for multiplex PCR to amplify the intended target loci of each genome from F_2_ progeny. Products in the amplified pools were modified by adding sequencing primer tags through secondary PCR. Modified pools were used to construct libraries for each individual. The effective concentration of libraries was determined by qRT-PCR. Libraries with more than 2 nM of the effective concentration were selected for sequencing by Paired-end 150 bp Method in the Illumina HiSeq system. After filtering unclean reads from raw reads obtained by sequencing, clean reads were obtained, which were mapped to the reference genome (ZS11-v20200127) using the MEM algorithm in BWA software. The mapping rate, coverage scope, coverage depth, and coverage uniformity of the reference genome were assessed.

### Linkage Map and QTL Analysis

Loci polymorphisms of the clean reads mapped to the reference were detected using GATK software (version 3.7), and single-nucleotide polymorphisms (SNPs) and insertions and deletions (InDels) were obtained to construct a linkage map of the F_2_ population according to the methods of Xie et al. (2010). QTLs were detected by a composite interval mapping method using QTL Cartographer software (version 1.17j). The LOD thresholds of the QTLs were determined by a 1,000 permutation test at a 95% confidence level.

### RNA-seq Analysis

RNA was extracted from the leaves of two parents (NTS57 and CY12) after treatment at low temperature for vernalization and used in the subsequent RNA-seq. Total RNA was extracted using TRIzol Reagent (Tiange Biotech, China) according to the manufacturer’s instructions. The library construction and sequencing were performed by Gene Denovo Biotechnology Co. (Guangzhou, China) on an Illumina HiSeqTM2500 platform. The raw sequences generated from the Illumina sequencing were filtered to remove the adaptor sequences and low-quality sequence reads from the data sets. Clean reads were then mapped to the *B. napus* reference genome using TopHat2 (version 2.0.3.12) (Kim et al., 2013). The gene expression level was calculated by the reads per kb per million reads. Genes with a fold change ≥ 2 and a *p*-value < 0.01 were filtered as DEGs using the edgeR package^1^. Enrichment analysis of the DEGs was accomplished based on the gene ontology (GO) database and Kyoto Encyclopedia of Genes and Genomes (KEGGs) database. Both GO terms and KEGG pathways with a *Q*-value of ≤ 0.05 were significantly enriched by DEGs. The clustered profiles with a *p*-value of ≤ 0.05 were considered to be significant profiles. The RNA-seq raw data have been submitted to the SRA of NCBI with the accession number Sra552729.

#### Total RNA Extraction and Real-Time Quantitative PCR

Total RNA in leaves was extracted as per the manufacturer’s instructions (TIANGEN Biotech (Beijing) Co., Ltd.), and RNA integrity was detected by the electrophoresis. The RNA was reverse-transcribed (PrimeScript™ RT Reagent Kit with gDNA Eraser, TaKaRa) to obtain single-stranded cDNA. In total, three replicates were performed for each sample. After measuring the concentration, the cDNA was stored at −20°C. The primers used for real-time quantitative PCR (qRT-PCR) were listed in Supplementary Table 1. qRT-PCR amplification reactions were performed using a LightCycler^®^96 RTPCR System (Roche, Basel, Switzerland) with SYBR qPCR Mix (Invitrogen, California, United States). *Actin-1* was used as an internal reference, and the relative expression of each gene was analyzed using the 2^–ΔΔCt^ method.

## Results

### Phenotypic Feature of Flowering Time

**FIGURE 1:**
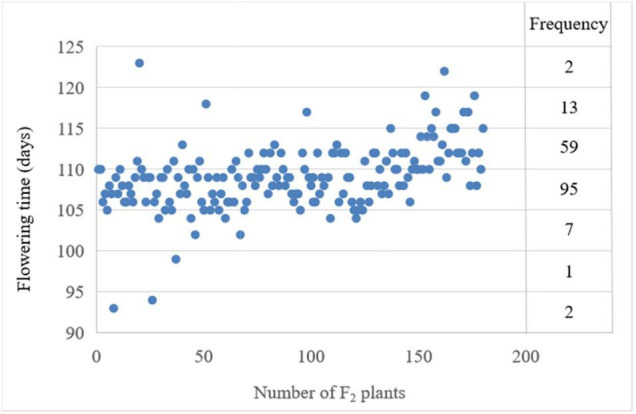
Frequency and distribution of the phenotypic value of the flowering time and frequency in the interval spanning 10 days were labeled on the right.

For the number of days from sowing to first flowering, i.e., flowering time, the phenotypic data showed significant differences between the two parents. The flowering time of the late-flowering parent NTS57 is 121 and 20 days later than the early-flowering parent CY12 (Table 1). The flowering time of F_2_ individuals ranged from 93 to 123 days, with an interval of 30 days. The flowering time of F_2_ plants exhibited normal distribution, showing features of quantitative hereditary traits. The broad sense heritability of FT trait among F_2_ progeny is 0.621 (Table 1). The flowering time of three individuals in the F_2_ population ranged from 2 to 8 days earlier than that of the early-flowering parent CY12, and that of two individuals was later than that of the late-flowering parent NTS57. These earlier- or later-flowering individuals are of higher value in the rapeseed breeding compared with the two parents (Figure 1).

**TABLE 1:** Statistical analysis of parents, F1, and F2 progeny for flowering time.

Parents Mean		F1	F2		
NTS57	CY12	Mean ± SD	Range	Mean ± SD	Heritability
121 ± 3.79	101 ± 2.52**	118 ± 4.44	93-123	109 ± 3.27	0.621

**Represents the extremely significant level between two parents.

### A Targeted Sequence of F_2_ Population and Variation Analysis

The mean coverage of the genome percentage and mean genome coverage depth of the parents and F_2_ families were 0.08% and 108.29×, respectively. After filtrating, low-quality reads from raw reads obtained by the targeted sequencing of the two parents and 178 F_2_ plants, there was an average of 308.56 thousand clean reads per plant, including 88.62 million bases. A total of 1,034 variants were obtained through comparison of the genotype of the two parents, including 946 SNPs, 88 InDels, 563 transitions (i.e., 282 A/G and 281 C/T), and 378 transversions. Among InDels, one- and two-base deletions/insertions were the most common. These variants were distributed equally throughout the genome.

### Construction of the Genetic Linkage Map

A total of 1,034 markers showing polymorphism between the two parents were used to construct a genetic linkage map, 1,017 of which were grouped into 19 linkage groups. The total length of the linkage map was 793.53 cM, and the average genetic distance between the two adjacent markers was 0.051 cM. Several markers lacking signs of recombination between adjacent markers were merged into one bin, and 268 bins were contained in this linkage map with an average genetic distance of 0.233 cM between the two adjacent bins. The linkage group with the longest genetic length was C06, which was also the linkage group with the largest number of bins (28). The linkage group with the lowest number of bins (2 bins containing 19 markers) was A10, and its genetic distance (6.43 cM) was the shortest (Table 2).

**TABLE 2:** Detailed information of the genetic map constructed in the F_2_ population.

Chr	Length (cM)	No. markers	No. bins	Marker interval (cM)	Bin interval (cM)	Max interval (cM)
A01	35.072	87	18	0.408	2.063	5.735
A02	33.136	29	8	1.183	4.734	13.881
A03	57.528	27	5	2.213	14.382	37.445
A04	33.901	43	12	0.807	3.082	10.371
A05	16.362	48	8	0.348	2.337	5.335
A06	57.659	29	8	2.059	8.237	31.912
A07	36.537	36	6	1.044	7.307	14.325
A08	43.029	25	8	1.793	6.147	38.078
A09	42.131	49	11	0.878	4.213	7.265
A10	6.430	19	2	0.357	6.430	6.430
C01	42.278	81	19	0.528	2.349	11.356
C02	41.460	110	24	0.380	1.803	6.131
C03	58.663	52	12	1.150	5.333	15.542
C04	49.281	99	26	0.503	1.971	9.789
C05	24.294	43	13	0.578	2.025	7.159
C06	59.922	78	28	0.778	2.219	8.697
C07	59.180	31	13	1.973	4.932	28.748
C08	59.043	62	22	0.968	2.812	7.066
C09	37.622	69	25	0.553	1.568	11.062
Whole	793.528	1,017	268	0.974	4.418	14.544
Mean	41.765	53.526	14.105	0.051	0.233	0.765

### QTL Mapping of Flowering Time

**FIGURE 2:**
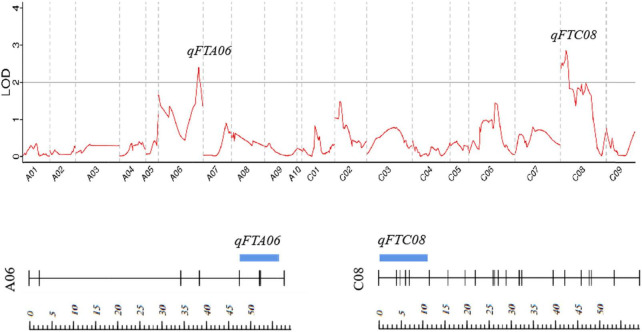
Distribution of the putative QTLs for flowering time on the genetic map. Strips represent QTLs detected, and their location and length represent their location of QTL on the linkage map and interval span. The scale represents the genetic distance (cM).

A total of two QTLs (qFTA06 and qFTC08) associated with the flowering time were detected on two chromosomes (A06 and C08) in the F_2_ population, with LOD values of 2.39 and 2.85, respectively. qFTA06 accounted for 19.3% of the phenotypic variation and was located on an 8.8 cM interval from 47.47 to 56.27 cM, mapping to the physical interval from 41.5 to 48.5 Mb on chromosome A06 of reference genome ZS11. qFTC08 accounted for 4.60% of the phenotypic variation and was located at the interval from 0.01 to 10.95 cM of the C08 linkage group (Table 3 and Figure 2).

**TABLE 3:** Detailed information of QTL for flowering time.

QTL	Chr	Pos(cM)	LOD	R^2^	Additive effect	Dominant effect	Confidence interval	Bin interval	Physical interval
*qFTA06*	A06	51.98	2.39	19.30%	8.22	1.42	47.47-56.27	c06b005-c06b008	41514735-48581582
*qFTc08*	C08	6.95	2.85	8.60%	–3.43	–0.48	0.01-10.95	C08b001-c08b006	859254-28988144

### Illumina Sequencing and Mapped Clean Reads

To verify transcription-level changes, six cDNA libraries were constructed using total RNA extracted from the leaves of two winter rapeseed varieties, NTS57 and CY12, treated at low temperature for 20 (t1) and 40 (t2) days and continuous room temperature (t0). The *de novo* transcriptome assembly and annotation were implemented. A Phred quality score of > 30 (Q30) was > 92%, and the guanine-cytosine (GC) content was consistently 48% for the four samples, indicating that the sequencing results were satisfactory. Raw sequences from cDNA libraries were filtered to obtain high-quality clean reads. After trimming and removing low-quality reads and ribosomal RNAs, 845.5 million clean reads remained and were used for quantitative analysis of gene expression. These clean reads were mapped to the reference *B. napus* genome using Tophat 2 software (2.1.1 versions), and 77.96–81.35% of the clean reads were mapped successfully to the genome, including 65.61–68.24% of the unique mapped reads and 12.35–13.11% of the multiple mapped reads. The remaining 18.65–22.04% of the reads were unmapped to the genome (Table 4). A total of 78,197 genes were detected across all the samples of both varieties, including 6,387 new genes and 71,810 known genes.

**TABLE 4:** Summary of the read numbers from the RNA-seq data for the four samples.

	Total reads	Unmapped reads		Unique mapped reads		Multiple mapped reads		Mapping ration
NTS57t0	144693890	29195649	20.18%	97189781	67.17%	18308460	12.65	79.82%
NTS57t1	155353088	30407162	19.57%	105202728	67.72%	19743198	12.71	80.43%
NTS57t2	128926068	26973068	20.92%	85748328	66.51%	16204672	12.57	79.08%
CY12t0	132109208	24637786	18.65%	90156636	68.24%	17314786	13.11	81.35%
CY12t1	128026106	28219780	22.04%	83998414	65.61%	15807912	12.35	77.96%
CY12t2	141845114	27246114	19.21	96320426	67.91%	18278574	12.89	80.79%

### Differences in Gene Expression Between Early- and Late-Flowering Varieties

**FIGURE 3:**
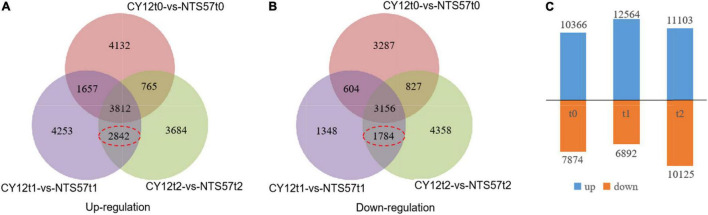
Gene expression profiles and the differentially expressed genes (DEGs) identified between early- and late-flowering genotypes. The Venn diagram shows the numbers of upregulated **(A)** and downregulated **(B)** DEGs between two varieties with the different flowering times under three temperature treatments and the number of overlaps. The number of DEGs is shown in **(C)**.

To identify the important genes related to flowering time, the difference in gene expression between two varieties, early-flowering CY12 and late-flowering NTS57, was analyzed. NTS57 seedlings were maintained at low temperature for 20 days and contained 19,456 significant DEGs, including 12,866 upregulated and 7,086 downregulated DEGs. NTS57 seedlings were maintained at low temperature for 40 days and contained 21,228 significant DEGs, including 11,103 upregulated and 10,125 downregulated DEGs (Figure 3). NTS57 seedlings were maintained at normal temperature and contained 10,820 DEGs, including 10,366 upregulated and 7,874 downregulated DEGs (| log2(TP/SP)| > 1 and *P* ≤ 0.001).

**FIGURE 4:**
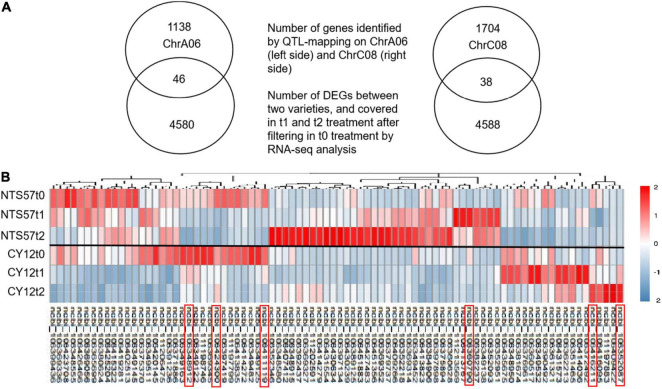
Overlap in the DEGs identified using RNA-seq and located in QTLs. **(A)** The Venn diagram shows the number of genes located in QTLs related to the flowering time, the number of DEGs in the two varieties at low temperature, and the number of overlaps. **(B)** Heatmap showing clustered gene expression in early- and late-flowering genotypes. Different colors represent different expression levels in the late-flowering variety NTS57 and the early-flowering variety CY12 under three temperature treatments. The candidate genes selected that relate to flowering time are marked with a red rectangle.

In total, 11,594 DEGs were common to both 20-day- and 40-day-exposed *B. napus*, including 6,654 upregulated and 4,940 downregulated DEGs. Of these, 4,626 DEGs (2,842 upregulated and 1,784 downregulated), were specific to low-temperature vernalization, and these were most likely the basis for variation in flowering time. Therefore, these 4,642 genes were used to further narrow the region containing QTLs related to the flowering time (Figure 4).

### Analysis of the Crossover Between the Candidates Identified by QTL Mapping and RNA-seq Reduced the Number of Responsible Genes

RNA-seq analysis was used to narrow down the candidate genes identified *via* QTL mapping and identify specific DEGs associated with vernalization at low temperature in early- and late-flowering *B. napus* genotypes. A total of 4,642 specific genes were identified by RNA-seq., and we speculate that some important genes affecting variations in flowering time were included in these gene groups. Two QTLs related to the flowering time, qFTA06 and qFTC08, were found on the chromosomes A06 and C08, respectively. A total of 2,926 genes were distributed in two QTL regions, including 1,194 genes in the qFTA06 region and 1,732 genes in the qFTC08 region. Crossover analysis was used to integrate QTL mapping and global transcriptomic data. In total, eighty-four DEGs in early- and late-flowering varieties at low temperature were found in two QTL regions related to the flowering time. Of these, 46 genes were located in the qFTA06 region, and 38 genes were located in the qFTC08 region. A total of fifty-six genes was upregulated and 28 genes were downregulated in the late-flowering genotype (Figure 4). In total, six genes were candidate genes affecting flowering and were involved in flower differentiation, flowering time, and floral organ formation. The IDs of the candidate genes and their annotations are listed in Table 5. *BnaC08G0115300ZS*, located in the qFTC08 region and encoding a rapid alkalinization factor (RALF32), belongs to a family of cysteine-rich plant peptide hormones that are involved in the multiple physiological and developmental processes such as growth and development of shoots, buds, anther, and pollen. RALF32 binds to CrRLK1L FERONIA (FER) to inhibit the development of the shoot apex (Abarca et al., 2021). *BnaC08G0356200ZS* encodes plant-specific Rop nucleotide exchange factor 6, which regulates many developmental processes such as the transition from vegetative growth to the reproduction of flowering plants through the auxin and ABA-signaling pathways (Zhang and McCormick, 2007; Kim et al., 2020). *BnaA06T0437200ZS* encodes a transcription activator GLK2-like (also called mitogen-activated protein kinase; MAPK) that belongs to the serine/threonine kinase family and is involved in the vernalization pathway in the cold signal perception and responsive networks (Gou et al., 2018; Xu and Chong, 2018; Chuang and Tan, 2019). *BnaC08G0010400ZS* encodes Cryptochrome 2 (CRY2), a conserved photoreceptor that accelerates flowering time by regulating the circadian clock (El-Din et al., 2003; Nefissi et al., 2011; Wu et al., 2021). *BnaA06T0363000ZS*, an ortholog of *At3G17800* that encodes a UV-B-induced protein with an unknown function, was significantly downregulated in two varieties of rapeseed during low-temperature vernalization. *BnaC08G0010400ZS* encodes a hypothetical protein that is a homolog of the flowering-promoting factor (FPF1) in *Arabidopsis*. FPF1 confers the promotion of flowering time by affecting auxin homeostasis.

**TABLE 5:** Candidate genes narrowed down by QTL-mapping and RNA-seq experiments and their annotations.

Candidate Gene	Regulation	Pfam_annnotation	Description
*BnaA06G0363000ZS*	Down	Protein of unknown function (DUF760)	UV-B-induced protein At3g17800, chloroplastic [Brassica rapa]
*BnaA06G0437200ZS*	Down	Myb-like DNA-binding domain	transcription activator GLK2-like [Brassica napus]
*BnaA06G0332400ZS*	Up	hypothetical protein	Flowering-promoting factor 1 OS = Sinapis alba GN = FPF1 PE = 2 SV = 1
*BnaC08G0115300ZS*	Down	Rapid ALkalinization Factor (RALF)	protein RALF-like 32 [Brassica oleracea var. oleracea] [Brassica oleracea]
*BnaC08G0356200ZS*	Up	PRONE (Plant-specific Rop nucleotide exchanger)	rop guanine nucleotide exchange factor 6 isoform X1 [Brassica napus]
*BnaC08G0010400ZS*	Down	DNA photolyase	cryptochrome-2 [Brassica napus];Cryptochrome-2 OS = Arabidopsis thaliana GN = CRY2 PE = 1 SV = 2

*The ‘down’ or ‘up’ indicate the downregulation or upregulation of gene expression at the low temperature relative to that at the normal temperature.*

#### Expression of Candidate Genes in the Early- and Late-Flowering Varieties

**FIGURE 5:**
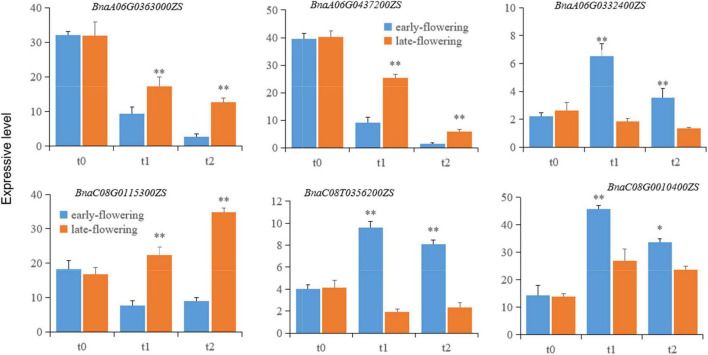
The expression of putative genes related to flowering time in early- and late-flowering *B. napus* varieties during vernalization. Each candidate gene is labeled in the upper part of each panel. t0, t1, and t2 represent unvernalization and vernalization treatments for 20 and 40 days. Single and double asterisks (*,^**^) indicate statistical significance relative to the control at *p* < 0.05 and *p* < 0.01, respectively. The error bars represent the standard variations.

To investigate variation in candidate gene expression, total mRNAs were extracted from leaves of early- and late-flowering varieties treated at low temperature for 20 (t1) and 40 (t2) days for vernalization and from unvernalized samples as a control (t0). The mRNA levels of the candidate genes were detected using qRT-PCR. The expression of *BnaA06TG0363000ZS, BnaA06T0437200ZS*, and *BnaC08T0115300ZS* was significantly lower in the early-flowering variety than in the late-flowering variety. Conversely, the expression of *BnaA06G0332400ZS, BnaC08T0356200ZS*, and *BnaC08G0010400ZS* was significantly higher in the early-flowering variety than in the late-flowering variety (Figure 5). This pattern was consistent at both the RNA-seq and mRNA levels, indicating that the RNA-seq results were reliable in this study.

## Discussion

As a sessile organism, winter rapeseed is exposed to a wide range of ambient cues and must adjust its growth and development according to this environmental variation. For overwintering or biennial crops, flowering at the proper time and in season is vital to avoid ambient damage and improve crop yield (Xu and Chong, 2018). In the northern region of China, winter rapeseed is planted in early autumn and flowers the following spring. Winter is long and frosty; therefore, strong winter rapeseed genotypes are required to avoid premature flowering (Liu et al., 2021). Simultaneously, the winter low temperatures provide sufficient cold for vernalization to initiate floral transition the following spring. In China, winter rapeseed is primarily distributed in the Yangtze River valley. No existing winter variety can survive in this cold and arid region. Fortunately, several strong winter varieties were bred by our laboratory, and these varieties have been planted successfully in cold and arid regions. Flowering time is one of the most important adaptive traits in the winter rapeseed. In previous studies, populations developed by crossing early- and late-flowering *B. napus* genotypes (e.g., RILs, DH, F_2_, F_2:3_ posterities) were used in QTL analyses of the flowering time. Several QTLs associated with flowering time have been reported, and all but three of these (Chr. A01, A09, and C01) are distributed on 16 chromosomes (Fletcher et al., 2015; Jian et al., 2019; Scheben et al., 2020; Xu et al., 2020; Song et al., 2021 and Supplementary Figure 1). Both minor QTLs with small phenotypic contributions and major QTLs accounting for > 10% of the phenotypic variation have been reported. For example, Li et al. (2018) identified 100 QTLs associated with flowering time using a DH population containing 348 lines. These included 25 major QTLs located on chromosomes A06, A02, A03, and C06 that explained > 10% of the phenotypic variation. Two QTLs associated with flowering time were located on chromosomes A10 and C06 in an F_2_ population (Li et al., 2018). In this study, two QTLs associated with flowering time were found in two regions on chromosomes A06 and C08 in F_2_ progeny developed by hybridizing strong winter genotype (NTS57) and spring genotype (CY12), respectively. One of these, qFTC08, was found in the 28.1 Mb interval of chromosome C08 and accounted for 8.6% of the phenotypic variation. The other, qFTA06, is a major QTL found in the 7.06 Mb interval of chromosome A06 and accounts for 19.3% of the phenotypic variation. QTL analysis identified molecular markers correlating with flowering time and allowed for the selection of oilseed rape based on the molecular markers.

RNA-seq was used to detect DEGs in strong winter and spring types during vernalization. A total of 4,626 DEGs, including 2,842 upregulated and 1,784 downregulated DEGs, were identified, and their expression differed only at low temperatures during vernalization and not at normal temperatures. Several important genes for flowering time may be among these, and their differential expression may be the molecular basis of variation in flowering time between two varieties of oilseed rape. In QTL regions, qFTA06 and qFTC08, 1,138 and 1,704 genes were included, respectively. To narrow down genes that are important for flowering time, QTL mapping and RNA-seq results were integrated. In total, 2,842 genes were identified by QTL mapping and 4,626 DEGs revealed by RNA-seq were crossed and 84 overlapping genes were identified. In a subsequent analysis of KO items and the KEGG pathway, six genes were identified as candidates involved in flowering.

The six genes included in the major flowering pathway regulate the circadian clock/photoperiod, vernalization, auxin and ABA signaling, and the MAPK cascade in the cold signal transduction pathway. *BnaC08G0115300ZS* encodes a plant peptide hormone involved in the differentiation of the shoot apex (Abarca et al., 2021). Shoot apices can differentiate into either flower buds and flowers or branch buds and branches. *BnaC08G0010400ZS* encodes a homolog of FPF1 that modulates flowering time (Wang et al., 2014), and overexpression of FPF1 causes early flowering (Ge et al., 2004). In rice, FPF1-like protein 4 (OsFPFL4) is a homolog of AtFPF1 that modulates auxin homeostasis to control flowering time (Guo et al., 2020). *BnaC08G0115300ZS* expression is higher in the late-flowering *B. napus* genotype than in the early-flowering genotype, suggesting that this gene suppresses flowering. *BnaC08G0356200ZS* encodes plant-specific Rop nucleotide exchange factor 6, which is involved in the regulation of the auxin and ABA-signaling pathway. The ABA-signaling pathway is involved in the regulation of photoperiod and circadian rhythms and promotes floral transition (de Montaigu et al., 2010). In our study, *BnaC08G0356200ZS* was upregulated and was higher in the early-flowering genotype, which implies that the upregulation of these two genes promotes oilseed rape flowering. *BnaA06G0437200ZS* encodes an ortholog of a transcription activator MAPK involved in the perception and transduction of the cold signal required for vernalization (Gou et al., 2018; Xu and Chong, 2018; Chuang and Tan, 2019). *BnaC08G0010400ZS* and *BnaA06T0363000ZS* regulate photoperiod and influence flowering (El-Din et al., 2003; Nefissi et al., 2011; Wu et al., 2021). In this study, these candidate genes were differentially expressed between early- and late-flowering genotypes during vernalization, which suggests that these candidate genes are important in regulating the floral translation of oilseed rape.

## Conclusion

In this study, a genetic linkage map was constructed based on 1,017 markers that merged into 268 bins covering 793.53 cM. Two QTLs were detected that were distributed on the two chromosomes, including one major QTL on chromosome A06 that accounted for 19.3% of the phenotypic variation. RNA-seq analysis identified 4,626 DEGs between early- and late-flowering genotypes during vernalization. Integrated analysis using QTL mapping and RNA-seq identified 84 DEGs in the QTL regions. Of these, six candidate genes were involved in the regulation of flowering time and played an important role in the flowering-related pathways.

## Data Availability Statement

The original contributions presented in the study are included in the article/Supplementary Material, further inquiries can be directed to the corresponding author/s.

## Author Contributions

ZL, XD, GZ, HL, XF, YW, and HT conceived the study. GZ, CX, XC, and HL developed the experimental populations. JW and JC completed the phenotyping, genotyping, and bioinformatic analyses. XD and GZ performed the linkage mapping. GZ completed the gene expression analyses. All authors contributed to the writing and/or editing of the manuscript and approved the final version of the manuscript.

## Conflict of Interest

The authors declare that the research was conducted in the absence of any commercial or financial relationships that could be construed as a potential conflict of interest.

## Publisher’s Note

All claims expressed in this article are solely those of the authors and do not necessarily represent those of their affiliated organizations, or those of the publisher, the editors and the reviewers. Any product that may be evaluated in this article, or claim that may be made by its manufacturer, is not guaranteed or endorsed by the publisher.

## Footnotes

1
http://www.r-project.org/
