# Supplementary material for: Integrate QTL Mapping and Transcription Profiles Reveal Candidate Genes Regulating Flowering Time in Brassica napus
Source: Front Plant Sci. 2022 Jun 28;13:904198. doi: 10.3389/fpls.2022.904198 (PMC9274139; doi:10.3389/fpls.2022.904198)
Supplement: Supplementary file 1 [file Image_1.pdf]

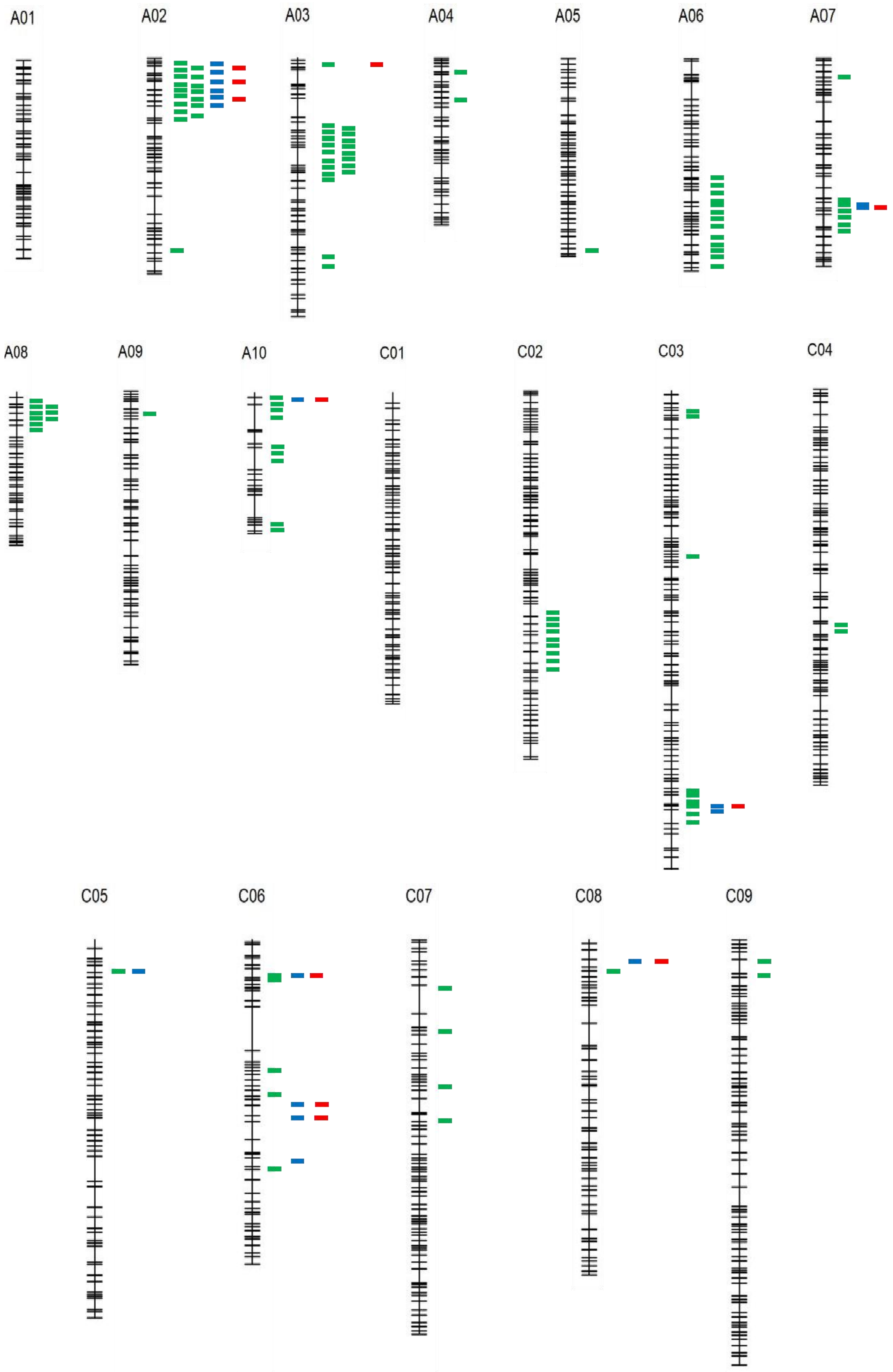

Fig. S1 sketch map shows distribution of QTLs, reported in main previous studies for flowering time , on chromosomes of *Brassica napus*. Green bars represent minor QTLs. Blue bars represent major QTLs. Red bars represent common QTLs.
