# Supplementary material for: Integrate QTL Mapping and Transcription Profiles Reveal Candidate Genes Regulating Flowering Time in Brassica napus
Source: Front Plant Sci. 2022 Jun 28;13:904198. doi: 10.3389/fpls.2022.904198 (PMC9274139; doi:10.3389/fpls.2022.904198)
Supplement: Supplementary file 2 [file Table_1.pdf]

Table S1 The primers used in real-time quantitative PCR to measure the expression of the key genes in cold signaling pathway

Candidate Gene	primer	
BnaA06G0363000ZS	F 5`-AGTAACTGCCAGACCGCGATA-3`	R 5`-CAGTCTTTCGAAGGCCAGAC-3`
BnaA06G0437200ZS	F 5`-CACAACGTAGCTAGCCATCTTC-3`	R 5`-CACTGTGGTATGTCGTCGGAG-3`
BnaA06G0332400ZS	F 5`-ATTTCTCTCCCTAAAGATTTCACCA-3`	R 5`-TACAGTCCGCAACACCCATAAGTTCT-3`
BnaC08G0115300ZS	F 5`-GAAACTAAAGGCATGGTGATGTC-3`	R 5`-TGGTACCTGTTCGACGG-3`
BnaC08G0356200ZS	F 5`-ACACCTTTGGGTTCAAGAGAATCGG-3`	R 5`-GAGTTTGCGATAATGGTTAGAGATG-3`
BnaC08G0010400ZS	F 5`-AGGACAGTTTTATCCAGGAAGGGCT-3`	R 5`-GCGACTGTTCTATCATTGGCTCAAG-3`
